# Healthcare-associated infective endocarditis: source of infection and burden of previous healthcare exposure

**DOI:** 10.1017/ash.2023.419

**Published:** 2023-09-08

**Authors:** Mika Halavaara, Kaisa Huotari, Veli-Jukka Anttila, Asko Järvinen

**Affiliations:** Department of Infectious Diseases, Inflammation Center, Helsinki University Hospital and University of Helsinki, Helsinki, Finland

**Keywords:** Nosocomial, non-nosocomial, hospital-acquired, *Enterococcus*, *Staphylococcus aureus*, bacteremia

## Abstract

**Objective:**

Prevention of healthcare-associated infective endocarditis (HAIE) is based on characterization of underlying factors. Our object was to describe the source of infection, microbiological etiology, and healthcare-related risk factors for HAIE.

**Design:**

Retrospective population-based study.

**Patients:**

Adult patients diagnosed with HAIE during 2013–2017 who resided in the study area in Southern Finland with adult population of 0.9 million.

**Results:**

Ninety-five HAIE episodes were included. Ten episodes were related to cardiac surgery. Of the remaining 85 episodes, 11 were classified as nosocomial (ie, acquired and diagnosed during ongoing hospitalization) and 74 as non-nosocomial HAIE. *Staphylococcus aureus* caused 45% of nosocomial episodes, but only 16% of non-nosocomial episodes (*P* = 0.039). Most common sources of infection in non-nosocomial HAIE were previous hospitalization (24%), dialysis (18%), and urologic procedures (15%). *Enterococcus* spp. caused 23% of non-nosocomial HAIE, and more than half of them were associated with urologic or gastrointestinal procedures. Two-thirds of the non-nosocomial HAIE patients had recent hospitalization or invasive procedure. We counted previous healthcare-related risk factors for IE and those who had two or more of them had higher in-hospital and one-year mortality.

**Conclusion:**

Our study indicates the importance of non-nosocomial acquisition of HAIE and *S. aureus* as the major pathogen in nosocomial episodes. Enterococcal infections dominate in non-nosocomial cases and further studies are needed to identify patients at risk for enterococcal IE after urological or gastrointestinal procedure.

## Introduction

Infective endocarditis (IE) is a rare, but devastating complication of health care.^1,2^ IE is classified as healthcare-associated (HAIE) when it is acquired in association with previous or ongoing hospitalization, previous invasive procedure, or if it occurs in a patient with a contact with health care.^3–6^ HAIE is associated with considerably worse prognosis compared to community-acquired IE.^4–7^ HAIE constitutes one-fourth to one-third of all IE cases, and its incidence is increasing.^4–8^ The increase is most likely attributable to aging of the population, increasing number of invasive diagnostic and therapeutic procedures, more common cardiac surgery, and widened diagnostic criteria for HAIE. HAIE might be preventable,^2^ but the causes behind it should be identified to direct preventive measures efficiently.

Important causes behind HAIE have been identified as catheter-related bacteremia, cardiac surgery, and urologic procedures.^4,6^ The evolution in clinical medicine might change these risk factors; thus, continuous surveillance is needed. Furthermore, the extent of prior healthcare exposure in HAIE is not well known in a population-based setting.

In our population-based study including all adult IE episodes diagnosed between the years 2013 and 2017 in Southern Finland, we found that one-third of all episodes and almost half of the episodes in patients who did not inject drugs were HAIE.^7^ The incidence of HAIE was 2.0/100,000 person-years in adult population. In this paper, we studied further the patients with HAIE to determine the source of infection, the associated microbiology, the extent, and impact of prior healthcare contact and differences between nosocomial and non-nosocomial acquisition of HAIE in a population-based level.

## Methods

This retrospective population-based study included all IE episodes which were diagnosed and treated in adult patients during the years 2013–2017 and who resided in the study area.^7^ All episodes met the modified Duke criteria for possible or definite IE.^9^ Detailed study protocol has been previously presented.^7^ Roughly 0.9 million adults resided in the study area, which consisted of six municipalities in Southern Finland. Acute care hospitals, three that belong to Helsinki University Hospital and two Helsinki city hospitals, are responsible for the treatment of severe infections, such as IE, in this area, and these hospitals were included in the study. Blood cultures are not taken outside the study hospitals, and thus, the probability is judged low that patients with IE were treated in other hospitals, non-hospital centers, or in the private sector and would not be identified in patient search of this study. However, some patients with bacteremia but not actively diagnosed or treated due to their poor medical condition may have been missed. The prevalence of methicillin-resistant *Staphylococcus aureus* (MRSA) in blood cultures has been low, and hence, only 8% of all IE episodes caused by *S. aureus* have been MRSA.^7^

**Figure 1.**
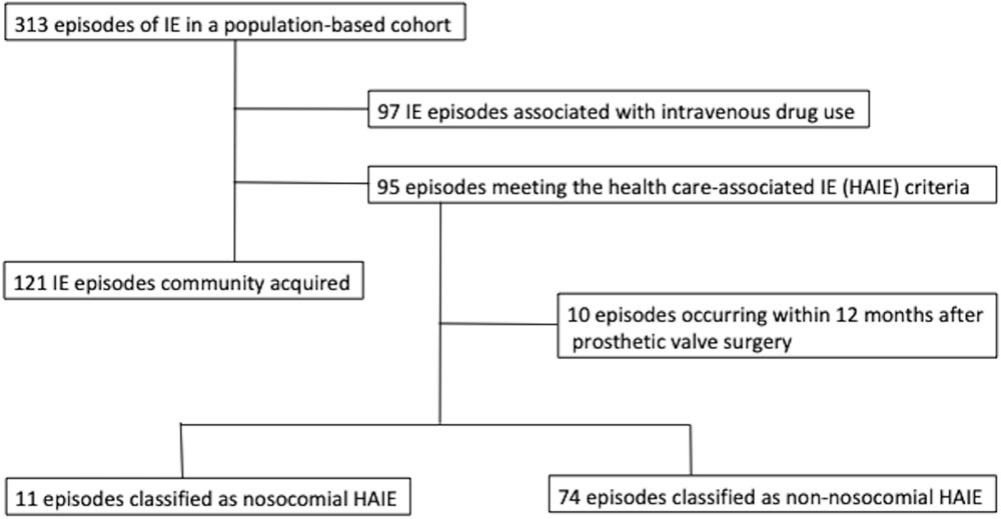
Flowchart of study design and the classification of infective endocarditis episodes in a population-based study cohort.

Patients were recognized from hospital electronic patient registry using ICD-10 diagnosis codes for IE. Data were gathered from electronic patient records and laboratory database. Data on IE episodes including the patient population and comparison between community-acquired IE and HAIE in persons who did not inject drugs are reported previously.^7^ In all, 313 IE episodes were included in the study population. Patients with no history of injection drug use and who met the criteria for HAIE (*n* = 95) were included in the present study for further analysis on comorbidities, the source of HAIE, previous healthcare contacts, and microbiology (Figure 1). Patients were followed for one year by review of their medical and laboratory records. If a patient had moved outside the study area, we may have missed the data on relapse, but we believe this number to be very low. Data on mortality were gathered from national register and are irrespective of place of residence.^7^ The research boards at the Inflammation Center, Helsinki University Hospital, and the City of Helsinki, approved the study protocol. Due to the retrospective nature of this study, informed consent was waived.

### Definitions

An episode of IE was defined as HAIE if any one of the following criteria were met: (1) onset of IE >48 hours after hospital admission or within six months after discharge from hospitalization (duration two days or more); (2) onset of IE within six months after a significant procedure known to be associated with bacteremia and an increased risk for IE and performed during hospitalization or ambulatory setting; (3) onset of IE within one month of extensive out-of-hospital contact with health care, defined as receipt of intravenous treatment, wound care, or vascular manipulations; or (4) residence in a nursing home or similar facility.^3,4,10,11^ A significant procedure associated with increased risk for IE includes several procedures, for example, cystoscopy, gastro- and colonoscopy, and intravascular procedures.^11^ Healthcare-related risk factor for IE means abovementioned connection with health care and any one of these risk factors was counted. In one episode, a patient could have multiple risk factors. In case of multiple risk factors, the most recent or most probable, as considered by the authors, was determined as the source of HAIE. IE occurring in a prosthetic valve within 12 months after initial operation was defined as HAIE. Dental procedures were not considered to be a criterion for HAIE because the causality is often very difficult to establish, and the data are not reliably retrievable.^4^ Episodes not related to cardiac surgery were further classified as nosocomial in case of IE onset and diagnosis of IE during ongoing hospitalization (48 hours after hospital admission) and non-nosocomial in any other cases. Thus, IE occurring and diagnosed after hospitalization was considered to be non-nosocomial. IE occurring in prosthetic valve within 12 months after initial valve operation (early PVE) has unique features and was thus analyzed separately. Early PVE is acquired perioperatively, and further classification to nosocomial or non-nosocomial acquisition was not feasible considering the definitions of these acquisitions used in this study (ie, nosocomial acquisition is limited to those IE episodes with onset and diagnosis during hospitalization).

### Statistical analysis

Data on categorical variables were presented as absolute numbers and percentages and were compared using Fisher’s exact test. Continuous variables were compared using Mann-Whitney test and were presented as median, range, and interquartile range (IQR), if appropriate. Multinomial logistic regression was used to compare patients with two healthcare risk factors and patients with three or more risk factors to patients with one risk factor. Odds ratios with 95% confidence intervals were calculated. A P-value <0.05 was considered statistically significant. Statistical analysis was conducted using SPSS, version 25.0 (IBM corp., Armonk, NY, USA).

## Results

In all, 95 HAIE episodes were identified (Figure 1). Ten cases were associated with cardiac valve surgery and analyzed separately. Of the remaining 85 episodes, 11 (12.9%) were nosocomial and 74 (87.1%) were non-nosocomial. The median age of patients was 69 years (range, 21–96), 73% (*n* = 69) were men, and 84% (*n* = 80) had two or more comorbidities.

Of all episodes, most common microbes responsible for HAIE were *Staphylococcus aureus* (21.1%, *n* = 20/95) and *Enterococcus* spp. (21.1%, *n* = 20/95). In 16 episodes (16.8%), viridans group streptococci (VGS) was the causative agent and in eight (8.4%) coagulase-negative *Staphylococcus* (CoNS). In 84 episodes (88.4%), the microbial etiology of HAIE was determined, and 81 episodes (85.3%) were blood culture positive. In three blood culture-negative cases, the microbial etiology of IE was confirmed by 16S rRNA analysis of cardiac valve sample obtained during surgery in one case and by bacterial culture results from another focus in two cases. Affected valves, complications, treatment, and outcome are described in detail elsewhere.^7^

### Nosocomial HAIE episodes (n = 11)

The source of infection could be determined in six episodes. Four of those were related to indwelling venous catheter: dialysis catheter in two episodes, central intravenous catheter, and peripheral intravenous catheter each in one episode. One episode was related to subcutaneous catheter used for analgesia and one to cardiac pacemaker installation.

*S. aureus* was responsible for five episodes, *E. faecalis* for two, and CoNS and *Candida glabrata* each for one episode. In two episodes, the microbiological etiology remained unknown. *S. aureus* was significantly more often responsible for IE in nosocomial cases (45.5%) compared to non-nosocomial cases (16.2%, *P* = 0.039; Table 1). Comparison of nosocomial and non-nosocomial HAIE episodes is shown in Table 1 with data on demographics, microbiology, complications, and outcome.

**Table 1.** Comparison between patients with nosocomial healthcare-associated infective endocarditis (HAIE) episodes and patients with non-nosocomial HAIE episodes

Variable	Nosocomial (*n* = 11)	Non-nosocomial (*n* = 74)	OR (95% CI)	*P*-value
**Patient characteristics**				
Age, years (median)	70	69		0.778
Age >60 years	9 (81.8)	53 (71.6)	1.78 (0.36–8.96)	0.719
Male gender	8 (72.7)	52 (70.3)	1.13 (0.27–4.66)	1.000
Two or more comorbidities^a^	11 (100)	60 (81.1)	1.18 (1.07–1.31)	0.198
History of previous IE	2 (18.2)	6 (8.1)	2.52 (0.44–14.42)	0.275
Known cardiac risk factor	5 (45.5)	34 (45.9)	0.98 (0.28–3.50)	1.000
Prosthetic valve	2 (18.2)	17 (23.0)	0.75 (0.15–3.78)	1.000
Diabetes	3 (27.3)	27 (36.5)	0.65 (0.16–2.67)	0.739
Hemodialysis	3 (27.3)	14 (18.9)	1.61 (0.38–6.84)	0.686
Malignancy	4 (36.4)	11 (14.9)	3.27 (0.82–13.08)	0.098
**Clinical characteristics and microbiology of IE**				
Left-sided IE	11 (100)	68 (91.9)	N/A	1.000
Blood culture positivity	9 (81.8)	65 (87.8)	0.62 (0.12–3.35)	0.630
*Staphylococcus aureus*	5 (45.5)	12 (16.2)	4.31 (1.13–16.41)	0.039
*Enterococcus* spp.	2 (18.2)	17 (23.0)	0.75 (0.15–3.78)	1.000
Viridans group str.	0	13 (17.6)	N/A	0.202
**Complications and outcome of IE**				
Stroke	0	9 (12.2)	N/A	0.597
Septic embolus^b^	5 (45.5)	26 (35.1)	1.54 (0.43–5.53)	0.520
ICU admission^c^	2 (18.2)	5 (6.8)	3.17 (0.52–18.20)	0.223
Surgical treatment	0	15 (20.3)	NA	0.200
In-hospital all-cause mortality	1 (9.1)	26 (35.1)	0.19 (0.02–1.52)	0.162
One-year all-cause mortality	3 (27.3)	36 (48.6)	0.40 (0.1–1.61)	0.214

Data are presented as number (%) unless otherwise indicated.

Abbreviations: HAIE, healthcare-associated infective endocarditis; OR, odds ratio; CI, confidence interval; IE, infective endocarditis; N/A, not applicable; str., streptococcus; ICU, intensive care unit.

a Comorbidities include atherosclerosis obliterans, atrial fibrillation, coronary artery disease, chronic kidney disease, chronic obstructive pulmonary disease, diabetes, hypertension, immunosuppressive treatment, liver cirrhosis, malignancy, heart valvulopathy.

b Other than stoke.

c Post-operative ICU admission not included.

### Non-nosocomial HAIE episodes (n = 74)

Previous hospital admission within six months, hemodialysis, and urologic procedures were the most common sources of infection (Table 2).

**Table 2.** Main source of infection and microbiology of 74 non-nosocomial healthcare-associated infective endocarditis (HAIE) episodes

Main source of infection	Number (%)	Microbes responsible for IE; *microbe* (number of cases)
Hospital admission within previous 6 months	18 (24.3)	*S. aureus* (4), *E. faecalis* (3), *E. faecium* (1), VGS (3), Other (6), Unknown (1)
Hemodialysis	13 (17.6)	*S. aureus* (4 [1 MRSA]), CoNS (3), VGS (3), *E. faecalis* (1), *S. lugdunensis* (1)
Urological procedure^a^	11 (14.9)	*E. faecalis* (6), VGS (2), CoNS (1), *E. faecalis* and CoNS (1), Unknown (1)
Gastrointestinal procedure^b^	5 (6.8)	*E. faecalis* (2), *E. faecium* (1), *S. agalactiae* (1), VGS (1)
Coronary angiography	3 (4.1)	CoNS (1), *Granulicatella adiacens* (1), VGS (1)
Other invasive procedure^c^	8 (10.8)	*S. aureus* (2), *E. faecalis* (2), *Gemella* spp. (1), VGS (1), Unknown (2)
Several possible procedures	7 (9.5)	*S. agalactiae* (2), *S. aureus* (1), *E. faecium* (1), G-group streptococci (1), CoNS (1), Polymicrobial (1)
Extensive healthcare contact or residency in a nursing home^d^	9 (12.2)	VGS (2), Group-G streptococci (2), *Aerococcus urinae* (1), *S. aureus* (1), *S. agalactiae* (1), *S. anginosus* (1), Unknown

Abbreviations: HAIE, healthcare-associated infective endocarditis; IE, infective endocarditis; VGS, Viridans group streptococci; CoNS, coagulase-negative streptococci;

From blood and other clinical culture samples, microbes were identified using routine diagnostic methods (including MALDI-TOF MS). 16S rRNA analysis was used if applicable (eg, cardiac valve samples) and also serology.^7,16^

a Transurethral resection of the prostate (TURP) five cases, cystoscopy 3, prostate biopsy 1, urinary catheter 1, orchiectomy 1

b Colonoscopy two cases, gastroscopy (with stricture dilatation and biopsies) 1, endoscopic retrograde cholangiopancreatography (ERCP) 1, abdominal surgery 1

c Peritoneal dialysis, mediastinoscopy, knee joint replacement, hip joint replacement, shoulder arthroscopy, surgery (removal of neck cyst), varicose vein operation, replacement of cardiac pacemaker battery; each one case.

d Residency in a nursing home or in a similar facility or extensive home health care six cases, wound care three cases.

*Enterococcus* spp. were responsible for 23% (*n* = 17) of cases, VGS for 17.6% (*n* = 13), *S. aureus* for 16.2% (*n* = 12), and CoNS for 9.5% (*n* = 7). In HAIE episodes associated with urologic or gastrointestinal procedures, *Enterococcus* spp. were the leading cause of HAIE (Table 2).

In Table 3, the previous healthcare contacts with a risk for IE are shown. In addition, while in 15 episodes a patient resided in a nursing home (or received similar care at home), this was the only HAIE criteria in only four of these cases.

**Table 3.** Healthcare-associated risk factors for IE in patients with non-nosocomial HAIE (*n* = 74). In each episode, a patient can have multiple risk factors

Variable	Number (%)
Hospitalization within previous 6 months	49 (66.2)
Hospitalization within previous 3 months	37 (50.0)
Two or more hospitalizations within previous 6 months	15 (17.6)
Invasive procedure within 6 months	51 (68.9)
Two or more invasive procedures within 6 months	21 (28.4)
Hemodialysis	13 (17.6)
Cardiac angiography	7 (9.5)
Non-cardiac angiography	4 (5.4)
Urologic procedures (cystoscopy, TURP, Prostate biopsy)	12 (16.2)
Gastroscopy	5 (6.8)
Colonoscopy	6 (8.1)
Puncture through skin or biopsy	6 (8.1)
Wound care (out-of-hospital)	8 (10.8)
Residence in a nursing home or receipt of specialized nursing care at home	15 (20.3)

Of the 74 non-nosocomial HAIE episodes, 61% of the patients (*n* = 45) had two or more healthcare-related risk factors for IE and they had significantly higher in-hospital and one-year all-cause mortality than those patients with only one risk factor (Table 4).

**Table 4.** Comparison of outcome in patients with non-nosocomial healthcare-associated infective endocarditis (*n* = 74) according to number of prior healthcare-associated risk factors

	Number of healthcare risk factor in one patient			One risk factor vs two risk factors		One risk factor vs three or more risk factors	
	One (*n* = 29)	Two (*n* = 21)	Three or more (*n* = 24)	OR (95% CI)	*P*-value	OR (95% CI)	*P*-value
In-hospital all-cause mortality	5 (17.2)	9 (42.9)	12 (50.0)	0.28 (0.08–1.01)	0.052	0.21 (0.06–0.73)	0.014
One-year all-cause mortality	6 (20.7)	12 (57.1)	18 (75)	0.20 (0.06–0.68)	0.010	0.09 (0.02–0.32)	<0.001

Data are presented as number (%) unless otherwise indicated.

Abbreviations: OR, odds ratio; CI, confidence interval.

### HAIE episodes associated with cardiac surgery

Ten HAIE episodes occurred within 12 months after valve surgery, which was considered to be the source of infection by definition. *S. aureus* and VGS were responsible for three episodes each and *Enterococcus faecalis* for one. In three episodes, the microbiological etiology could not be determined. Half of HAIE episodes in this group were operatively treated. All patients survived the hospitalization for HAIE, but two died within the first year after HAIE diagnosis.

## Discussion

The results of the present study highlight the importance of non-nosocomial acquisition of HAIE: of the 85 cases not related to cardiac surgery, 87% were non-nosocomial. Direct comparison of these proportions to previous studies is difficult due to differences in HAIE criteria and inclusion criteria between the studies, but previous studies have similarly observed the growing importance of non-nosocomial acquisition of HAIE accounting for 46%–67% of all HAIEs.^5,12^ In a register-based survey from California and New York State, non-nosocomial HAIE increased whereas nosocomial HAIE decreased during the study period years 1998–2013.^12^ Taken together, these data indicate that it is increasingly important to find non-nosocomial cases and that preventive measures targeting these risk factors have become most important.

In their work, Ben-Ami and colleagues proposed that the definition of HAIE should also include IE episodes occurring within six months after hospital discharge instead of three months.^10^ The present study, as well as two studies from Spain, applied this six-month criterion.^4,6^ The other Spanish study did not, however, include episodes in patients with extensive healthcare contact (eg, wound care) or residence in a nursing home,^4^ as proposed by Friedman and colleagues^3^ and adopted by some other studies^5,8^ and used in the present study. Ambulatory procedures associated with IE were included as HAIE in our study, and in other studies,^4,6^ but not systematically in all previous studies on HAIE.^5,8^ For future studies and for everyday, surveillance universal criteria for HAIE that would include change to more ambulatory care and increasing elderly care should be adopted.

Previous hospitalization was observed in two-thirds of non-nosocomial HAIE cases, and it was found a major risk factor for HAIE. Non-nosocomial cases seemed to concentrate in those patients that had multiple hospital admissions, multiple healthcare-associated risk factors, or previous invasive procedure, which were all recognized in two-thirds of cases.

Vascular manipulations have been the source of infection in HAIE episodes in half of the episodes.^4,6^ Prevention of catheter-related bloodstream infections (BSI) is one of the most important preventive measures of healthcare-associated infection. Quality improvement interventions, such as bundles and checklists, have been shown to reduce the risk of central line-associated BSIs.^13^ In our material, half of nosocomial cases were due to *S. aureus,* and indwelling venous catheter was recognized as a source of infection in 4/11 episodes. However, *S. aureus* was found only in 16% of nosocomial cases. In contrast, equal shares of *S. aureus* as etiology in nosocomial and non-nosocomial groups have previously been reported.^5,6^

As noted, the source of infection in patients with HAIE has been analyzed only in two recent studies.^4,6^ In a single-center study from Spain including 83 episodes of HAIE, hospital admission within previous six months was the only identifiable source of infection in 24.1% of episodes.^4^ This is in agreement with our results (24.3%) and points out its importance as a risk factor for HAIE. In addition, in one-fifth of episodes the source of infection was hemodialysis, as in our work. In approximately ten percent of cases, the source of infection was cardiac surgery both in the Spanish and our study. In our work, 15% of HAIE episodes had a urologic source of infection. Previous studies have reported them in 6%^4^ or 14%^6^ of HAIE episodes.

Enterococcal IE is a growing clinical entity which importance is well recognized due to difficulty in its management.^14^ In our material, *Enterococcus* spp. was the leading cause of IE in patients with non-nosocomial HAIE (23%) and most of these patients had undergone urologic or gastrointestinal procedures, a finding that should be noted when considering the empiric antibiotic treatment of HAIE in these scenarios. European Society of Cardiology does not recommend routine antibiotic prophylaxis for all patients who undergo gastrointestinal or urinary tract invasive procedure.^15^ They, however, do recommend that a patient who is at a high risk for IE and who receives antimicrobial therapy in this scenario, this therapy should have anti-enterococcal activity. Results from our study endorse this recommendation. In addition, in our hospital these data have led to addition of ampicillin to ciprofloxacin (previously ciprofloxacin alone) for prophylaxis in transurethral resection of prostate.

As BSI is a precursor for IE, all measures used in prevention of BSI will lead to reduction in IE eventually. However, although healthcare association predisposes to IE by procedures causing bacteremia, it should be noted that one-third of all patients did not have apparent invasive procedure performed. The mechanism behind IE in these cases is not clear but might be related to less invasive procedures that might cause transient bacteremia involving nosocomial microbes. Clinician should be aware of possible presence of IE also in these cases, which would enable an earlier diagnosis of IE and better prognosis.

There are important limitations in the interpretation of the results of this study. Due to the retrospective nature of this study, data are derived from medical patient records. Thus, we may have missed procedures that were not clearly recorded. Data on intravenous catheters as a source of infection were based on clinical notes by the treating clinicians, and catheters may have been missed. The findings of this study may not be generalized to a setting with higher rates of microbial resistance.

Our study points out that most episodes of HAIE were non-nosocomial, and previous hospitalization was the single most common source. In nosocomial HAIE, *S. aureus* was the dominant pathogen. Most HAIE associated with urological procedures were caused by *Enterococcus* spp., which might be an object for further studies to identify patients at risk for it. When previous healthcare-related risk factors were counted, the in-hospital and one-year mortality was significantly increased with more risk factors.
